# Clinical implications of *TP53* mutations in myelodysplastic syndromes treated with hypomethylating agents

**DOI:** 10.18632/oncotarget.7290

**Published:** 2016-02-09

**Authors:** Koichi Takahashi, Keyur Patel, Carlos Bueso-Ramos, Jianhua Zhang, Curtis Gumbs, Elias Jabbour, Tapan Kadia, Michael Andreff, Marina Konopleva, Courtney DiNardo, Naval Daver, Jorge Cortes, Zeev Estrov, Andrew Futreal, Hagop Kantarjian, Guillermo Garcia-Manero

**Affiliations:** ^1^ Department of Leukemia, The University of Texas MD Anderson Cancer Center, Houston, TX, USA; ^2^ Department of Hematopathology, The University of Texas MD Anderson Cancer Center, Houston, TX, USA; ^3^ Department of Genomic Medicine, The University of Texas MD Anderson Cancer Center, Houston, TX, USA; ^4^ Department of Hematology and Oncology, Graduate School of Medicine, Kyoto University, Kyoto, Japan

**Keywords:** TP53, myelodysplastic syndromes, hypomethylating agents

## Abstract

We screened *TP53* mutations in 168 MDS patients who were treated with HMA and evaluated predictive and prognostic value of *TP53* mutations. Overall response to HMA was not different based on *TP53* mutation status (45% vs. 32% in *TP53*-mutated and wild type [WT], respectively, P = 0.13). However, response duration was significantly shorter in *TP53*-mutated patients compared to WT patients (5.7 months vs. 28.5 months, P = 0.003). Longitudinal analysis of *TP53* mutations after HMA showed that *TP53* mutations almost always persisted at times of disease progression. *TP53*-mutated patients showed significantly worse overall survival (OS) compared to WT patients (9.4 months vs. 20.7 months, P <0.001). Further, *TP53* mutations distinguished prognosis in the subgroup of patients with complex karyotype and Revised International Prognostic Scoring System (IPSS-R) defined very high-risk disease. Multivariate analysis showed that *TP53* mutation status is significantly prognostic for OS after adjusting prognostic effect from other factors. The current study provides evidence that *TP53* mutations are independently prognostic in MDS patients treated with HMA. While *TP53*-mutated MDS patients initially respond well to HMA, their duration of response is significantly shorter than WT patients. Novel strategies to improve duration of response in *TP53*-mutated MDS are urgently needed.

## INTRODUCTION

*TP53* is a tumor suppressor gene that encodes the p53 protein, which acts as a transcription factor and induces cell cycle arrest, apoptosis, and cellular senescence. [1] Mutations in the coding sequence of *TP53* gene are one of the most common mechanisms of p53 deregulation, and they can be detected in more than 50% of all cancers. [2]

In myelodysplastic syndromes (MDS), *TP53* mutations are detected in approximately 5-20% of cases when modern deep sequencing methods are used. [3-8] Previous studies have consistently shown that *TP53* mutations are associated with higher-risk MDS, therapy-related disease, complex cytogenetics (including chromosome 5, 7 and 17 abnormalities), and poor overall survival. [4, 7-10] With regards to treatment response, *TP53* mutations are associated with resistance to cytarabine-based chemotherapy in MDS and acute myeloid leukemia (AML). [11, 12] In recent years, hypomethylating agents (HMA) such as 5-azacitidine or decitabine, has become the standard of care in higher risk MDS patients. [13, 14] However, it is not well understood whether *TP53* mutation status also predicts resistance to HMA therapy in patients with MDS. Further, while prognostic impact of *TP53* mutations has been well described, because of the strong correlation with other poor prognostic factors, such as complex karyotype, the independent prognostic value of *TP53* mutations is not clearly understood.

To better understand independent prognostic and predictive value of *TP53* mutations in the context of HMA therapy, we sequenced *TP53* in a large cohort of MDS patients who were treated with HMA therapy.

## RESULTS

### Patient characteristics

Clinical characteristics of the 168 patients at the time of sequencing are listed in Table 1. More than half of the patients were classified as having RAEB and 11% of them were RAEB-T. Compared to other published MDS genomics studies, our cohort included fewer patients with refractory anemia with ringed sideroblasts (RARS). [7, 10, 22] Majority of the patients had higher risk MDS and 56% of the patients were classified as high or very high risk by IPSS-R.

**Table 1:** Baseline clinical characteristics of the 168 patients with MDS who were screened for TP53 mutation and were treated with HMA therapy

Characteristics	N or median (% or range)
**Median age, range, y**	67 (17-89)
**Female**	64 (38)
**Pathological classification**	
**5q- syndrome**	2 (1)
**RA**	16 (9)
**RCMD**	27 (16)
**RCMD-RS**	1 (<1)
**RAEB-1**	37 (22)
**RAEB-2**	32 (19)
**RAEB-T**	19 (11)
**RARS**	3 (2)
**MDS-U**	1 (<1)
**MDS/MPD**	1 (<1)
**CMML-1**	21 (13)
**CMML-2**	8 (5)
**Therapy-related MN**	40 (24)
**Median WBC count, range, × 10^9^/L**	3.2 (0.6-162.0)
**Median ANC count, range, × 10^9^/L**	1.3 (0.0-103.7)
**Median HGB count, range, g/dL**	9.3 (6.0-15.8)
**Median PLT count, range, × 10^9^/L**	62 (2-655)
**Median BM blast count, range, %**	7 (0-29)
**Normal karyotype**	54 (32)
**Complex karyotype**	48 (29)
**Monosomal karyotype**	43 (26)
**Deletion 17p/monosomy 17**	15 (9)
**IPSS-R cytogenetic risk category**	
**Very good**	12 (7)
**Good**	53 (32)
**Intermediate**	51 (30)
**Poor**	3 (2)
**Very Poor**	47 (28)
**Unknown**	2 (1)
**Overall IPSS-R risk**	
**Very good**	9 (5)
**Good**	18 (11)
**Intermediate**	44 (26)
**High**	41 (24)
**Very high**	53 (32)
**Unknown**	3 (2)

Abbreviations. RA: refractory anemia, RCMD: refractory cytopenia with multilineage dysplasia, RCMD-RS: refractory cytopenia with multilineage dysplasia with ringed sideroblast, RAEB: refractory anemia with excess blast, RARS: refractory anemia with ringed sideroblast, MDS-U: myelodysplastic syndromes unclassified, MDS/MPD: myelodysplastic syndromes/myeloproliferative disease, MN: myeloid neoplasia, WBC: white blood cell, ANC: absolute neutrophil cells, HGB: hemoglobin, PLT: platelet, BM bone marrow, IPSS-R: Revised International Prognostic Scoring System.

* complex karyotype is defined as having > 3 chromosomal abnormalities.

† monosomal karyotype is defined as having at least 2 autosomal monosomies or a single autosomal monosomy associated with at least one structural chromosomal abnormality.

†† IPSS-R cytogenetic risk stratification is described as previously. [17]

### Landscape of *TP53* mutations

**Figure 1:**
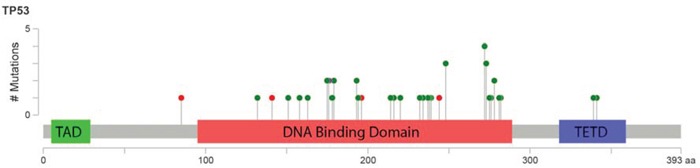
Lollipop figure of *TP53* mutations detected in 168 patients with MDS and CMML Green dots indicate missense mutations and red dots indicate nonsense mutations. The figure for was created using cBioPortal website (http://www.cbioportal.org/).

In total, 45 *TP53* mutations were detected in 38 patient samples (23%). Thirty-one patients had single *TP53* mutations, and 7 had double *TP53* mutations identified. Eighty-seven percent of the detected mutations were missense, 9% were nonsense, and 4% were frameshift indels. Among the missense and nonsense mutations, 69% were transition and 31% were transversion, and C/G>T/A alteration was most common. Mutations in *TP53* were predominantly detected in the core DNA-binding domain (93%), and only 2 mutations were detected in the tetramerization domain (Figure 1). The most frequently mutated codon was codon 272 (9%), followed by codons 273 (7%) and 248 (7%). Ninety-four percent of the detected missense mutations were predicted to be non-functional for transcriptional activity. [23] Ninety-five percent of them were predicted to be deleterious according to the SIFT algorithm. [24] The median variant allele frequency (VAF) of the *TP53* mutations was 35.4% (range: 8.9-93.3), and 95% of the detected *TP53* mutations had VAF ≥ 10% (Supplemental Figure 1).

### Correlation with other mutations

**Figure 2:**
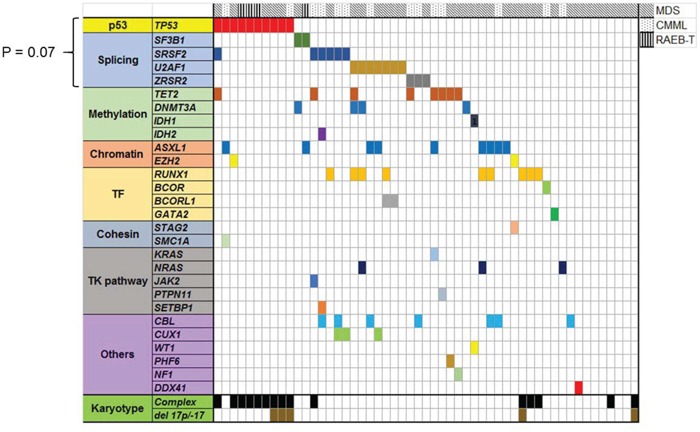
Landscape of well characterized myeloid driver mutations in 53 MDS/CMML patients whose bone marrow samples were sequenced by WES *TP53* mutated cases had less co-occuring mutations. *TP53* mutation and splicing gene mutations had trend to be mutually exclusive (P = 0.07).

For the patients whose WES data were available (N = 53), we investigated degree of co-occurrence and mutual exclusivity with other previously well-characterized myeloid driver mutations (Figure 2). Seven of 10 *TP53* mutated cases did not have any other co-occurring driver mutations, and this is consistent with other genomic studies that *TP53* mutated cases carry few co-occurring mutations. [3, 6, 10] Although statistically not significant, *TP53* mutation tended to be mutually exclusive to mutations in one of the RNA splicing pathway genes (*U2AF1, SRSF2, SF3B1, and ZRSR2*, P = 0.07).

### *TP53* mutations and clinical characteristics

Table 2 compares clinical characteristics of MDS patients based on *TP53* mutational status. By pathological classification, patients with RAEB-T had the highest frequency of *TP53* mutations (47%). *TP53* mutations were more frequently detected in therapy-related disease than in *de novo* disease. Patients with *TP53* mutations were also significantly more neutropenic, thrombocytopenic, and had higher bone marrow blasts at presentation. Furthermore, *TP53* mutation status was significantly associated with complex and monosomal karyotypes and 17p deletion / monosomy 17. Thirteen patients had both TP53 mutations and 17p deletion / monosomy 17. The majority of the *TP53* mutations were detected in patients with IPSS-R high or very high-risk disease.

**Table 2:** Comparison of clinical characteristics between TP53 mutated patients and wild type (WT) patients

	TP53 mutated	TP53 WT	
	N = 38 (%)	N = 130 (%)	P value
**WHO classification**			
**5q- syndrome**	1 (3)	1 (<1)	NA
**RA**	3 (8)	13 (1)	NA
**RCMD**	2 (5)	25 (19)	NA
**RCMD-RS**	0 (0)	1 (<1)	NA
**RAEB-1**	10 (26)	27 (21)	NA
**RAEB-2**	10 (26)	22 (17)	NA
**RAEB-T**	9 (24)	10 (8)	NA
**RARS**	1 (3)	2 (2)	NA
**MDS-U**	0 (0)	1 (<1)	NA
**MDS/MPD**	0 (0)	1 (<1)	NA
**CMML-1**	1 (3)	20 (15)	NA
**CMML-2**	1 (3)	7 (5)	NA
**Therapy-related MN, (%)**	16 (40)*	24 (60)*	0.003
**De novo disease, (%)**	22 (17)^†^	106 (83)^†^	
**Median WBC count, range, × 10^9^/L**	2.9 (1.0-29.5)	3.7 (0.6-162.0)	0.02
**Median ANC count, range, × 10^9^/L**	0.9 (0.05-21.5)	1.4 (0.0-103.7)	0.02
**Median HGB count, range, g/dL**	9.3 (6.8-12.8)	9.4 (6.0-15.8)	0.76
**Median PLT count, range, × 10^9^/L**	47 (9-290)	73 (2-655)	0.008
**Median BM blast count, range, %**	10 (0-29)	6 (0-30)	0.006
**Cytogenetics**			
**Complex karyotype**	32 (84)	16 (12)	<0.001
**Deletion 17p/−17**	13 (34)	2 (2)	<0.001
**Monosomal karyotype**	33 (87)	10 (8)	<0.001
**IPSS-R**			
**Very Low**	1 (3)	8 (6)	<0.001
**Low**	0 (0)	18 (14)	
**Intermediate**	1 (3)	43 (33)	
**High**	5 (13)	36 (28)	
**Very high**	30 (79)	23 (18)	
**Unknown**	1 (3)	2 (2)	

Abbreviations. RA: refractory anemia, RCMD: refractory cytopenia with multilineage dysplasia, RCMD-RS: refractory cytopenia with multilineage dysplasia with ringed sideroblast, RAEB: refractory anemia with excess blast, RARS: refractory anemia with ringed sideroblast, MDS-U: myelodysplastic syndromes unclassified, MDS/MPD: myelodysplastic syndromes/myeloproliferative disease, MN: myeloid neoplasia, WBC: white blood cell, ANC: absolute neutrophil cells, HGB: hemoglobin, PLT: platelet, BM bone marrow, IPSS-R: Revised International Prognostic Scoring System.

* Denominator is total number of therapy-related MN.

† Denominator is total number of de novo disease.

### Treatment response to HMA therapy

Complete response (CR) and overall response (OR) were observed in 49 patients (29%) and 57 patients (34%), respectively. Table 3 summarizes the association between various clinical characteristics and response to HMA therapy. Patients with thrombocytopenia at baseline had a significantly worse OR rate than patients with higher platelet count (23% versus 41%, P = 0.01). There was a non-significant trend toward better OR rate in patients with neutropenia than patients with higher neutrophil count (43% versus 28%, P = 0.06). No significant difference was observed in response rate by ages, type of therapy, extent of anemia, IPSS-R risk groups, and cytogenetic abnormalities. The HMA response rates were similar between patients with *TP53* mutations and WT *TP53* (*TP53* mutated vs. WT, CR: 34% vs. 27%, P = 0.38, OR: 45% vs. 32%, P = 0.13). Response rate was not different by *TP53* mutation status in patients sub-grouped by treatment types (SOC HMA versus HMA combination with investigational agents) (Supplemental Table 3). Correlations between treatment response and other myeloid driver mutations are described in Supplemental Table 4. In patients who were tested for *TET2* mutation (N = 79), there was no significant difference observed in response to HMA therapy by *TET2* mutation status (*TET2* mutated vs. WT, CR: 17% vs. 36%, P = 0.10, OR: 22% vs. 38%, P = 0.18). We also tested response rate based on both *TET2* and *ASXL1* mutation status because previous publication suggested high response rate in *TET2* mutated but *ASXL1* WT patients. [3] However, we did not see significant difference in response rate based on *TET2* and *ASXL1* mutation status (*TET2* mutated/*ASXL1* WT vs. other, CR: 20% vs. 34%, P = 0.23, OR: 27% vs. 36%, P = 0.36). In the current cohort, patients with mutation in *RAS* (*KRAS* and/or *NRAS*) had significantly worse response to HMA therapy (*RAS* mutated vs. WT, CR: 8% vs. 31%, P = 0.06, OR: 8% vs. 36%, P = 0.03). Although statistically not significant, *DNMT3A* mutation and mutation in one of the splicing pathway gene were associated with trend toward worse OR to HMA (Supplemental Table 4).

**Table 3:** Various clinical factors including TP53 mutation status and response to HMA therapy

Variables	Number	CR rate (%)	P value	OR rate (%)	P value
**All patients**	168	49 (29)	NA	57 (34)	NA
**Age < 70**	102	34 (33)	0.14	39 (38)	0.14
**Age ≥ 70**	66	15 (23)		18 (27)	
**MDS (including RAEB-T)**	139	40 (29)	0.81	47 (34)	0.95
**CMML**	29	9 (31)		10 (35)	
**WHO classification**			0.14		0.06
**5q- syndrome**	2	1 (50)		1 (50)	
**RA**	16	3 (19)		3 (19)	
**RCMD**	27	5 (19)		5 (19)	
**RCMD-RS**	1	0 (0)		0 (0)	
**RAEB-1**	37	11 (30)		14 (38)	
**RAEB-2**	32	7 (22)		9 (28)	
**RAEB-T**	19	10 (53)		11 (58)	
**RARS**	3	2 (67)		2 (67)	
**MDS-U**	1	0 (0)		1 (100)	
**MDS/MPD**	1	1 (100)		1 (100)	
**CMML-1**	21	5 (24)		6 (29)	
**CMML-2**	8	4(50)		4 (50)	
**Therapy-related MN**	128	38 (30)	0.79	44 (34)	0.83
**De novo disease**	40	11 (28)		13 (33)	
**ANC ≥ 0.8 × 10^9^/L**	109	26 (24)	0.06	31 (28)	0.06
**ANC < 0.8 × 10^9^/L**	58	22 (38)		25 (43)	
**HGB ≥ 8 g/dL**	148	44 (30)	0.66	52 (35)	0.37
**HGB < 8 g/dL**	20	5 (25)		5 (25)	
**PLT ≥ 50 × 10^9^/L**	102	35 (34)	0.07	42 (41)	0.01
**PLT < 50 × 10^9^/L**	66	14 (21)		15 (23)	
**BM blast ≤ 10%**	111	29 (26)	0.17	33 (30)	0.12
**BM blast > 10%**	55	20 (36)		23 (42)	
**Cytogenetics**					
**Non complex**	114	34 (30)	0.93	39 (34)	0.88
**Complex**	48	14 (30)		17 (35)	
**Non-monosmal**	119	34 (29)	0.62	39 (33)	0.42
**Monosomal**	43	14 (33)		17 (40)	
**IPSS-R**					
**Very Low/Low/Int**	72	19 (26)	0.60	23 (32)	0.74
**High/Very high**	93	28 (30)		32 (34)	
**Therapy types**					
**SOC Aza/DAC**	78	22 (28)	0.88	25 (32)	0.71
**HMA+investigational**	90	27 (30)		32 (35)	
***TP53*-mutated**	38	13 (34)	0.38	15 (45)	0.13
***TP53* WT**	130	35 (27)		41 (32)	

Abbreviations. CR: complete response, OR: overall response, RA: refractory anemia, RCMD: refractory cytopenia with multilineage dysplasia, RCMD-RS: refractory cytopenia with multilineage dysplasia with ringed sideroblast, RAEB: refractory anemia with excess blast, RARS: refractory

### Time to response and response duration in *TP53* mutated patients

**Figure 3:**
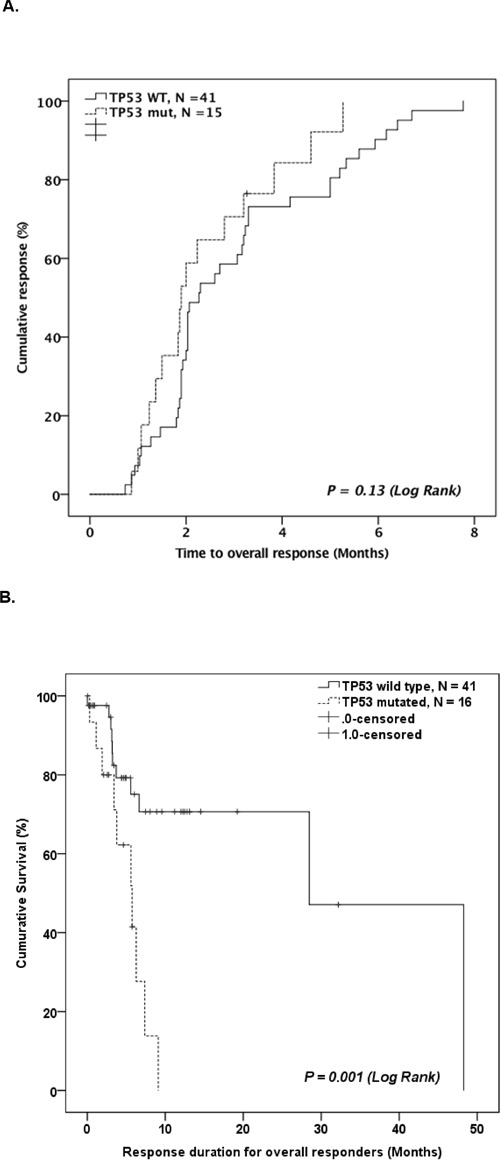
**A.** Kaplan-Meier curve comparing time to best response for *TP53* mutated patients and *TP53* WT patients who responded to HMA therapy. **B.** Kaplan-Meier curve comparing duration of response for *TP53* mutated patients and *TP53* WT patients who responded to HMA therapy.

For responders, there was no difference in time to response between *TP53*-mutated patients and WT patients (1.9 month vs. 2.3 month in *TP53*-mutated and WT patients, respectively, P = 0.08; Figure 3A). However, *TP53*-mutated patients had significantly shorter response duration compared to WT patients (5.7 months vs. 28.5 months in *TP53*-mutated and WT patients, respectively, P = 0.003, Figure 3B).

### Survival outcome

**Figure 4:**
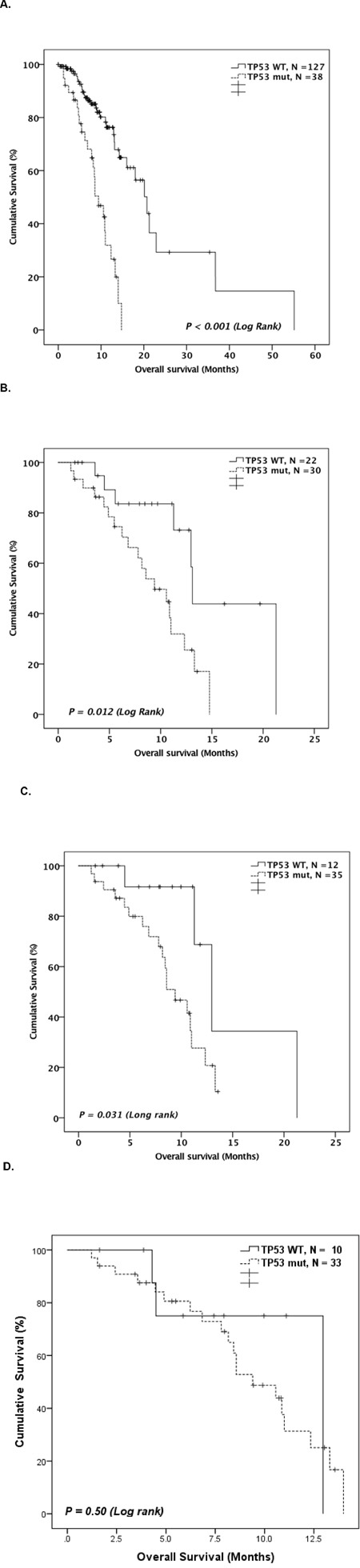
**A.** Kaplan-Meier curve comparing OS of *TP53* mutated patients and *TP53* WT patients. Kaplan-Meier curve comparing overall OS of *TP53* mutated patients and *TP53* WT patients among. **B.** patients with IPSS-R high or very high risk (N = 52). **C.** patients with complex karyotypes (N = 47), **D.** patients with monosomal karyotypes (N = 43).

The median OS of the studied patients was 14.8 months (95% CI: 11.8-17.7 months). *TP53* mutated patients had significantly worse OS compared to WT patients in both entire cohort (median 9.4 months [95% CI: 6.9-11.9] vs. 20.7 months [95% CI: 16.4-25.0], P <0.001; Figure 4A) and in patients subgrouped by treatment type (Supplemental Figure 2). *TP53* mutation status identified a distinct prognostic group of patients among the higher-risk patient groups. In the IPSS-R very high-risk group, *TP53*-mutated patients had significantly worse OS compared to WT patients (Figure 4B). Further, in a group of patients who had complex karyotypes, survival of *TP53* mutated patients was significantly worse than that of WT patients (Figure 4C). On the other hand, *TP53* mutation status did not differentiate survival outcome among monosomal karyotype patients (Figure 4D). Other clinical and mutational factors that were significantly prognostic to OS in univariate analysis are therapy-related disease, complex karyotype, monosomal karyotype, and IPSS-R high or very high risk group (Supplemental Table 5).

Multivariate analysis considering variables IPSS-R high and very high-risk (vs. others), *TP53* mutation (vs. WT), complex karyotype (vs. others), monosomal karyotype (vs. others), and therapy-related disease (vs. de novo) were conducted. Because complex karyotype and monosomal karyotype are strongly correlated, they were tested in separate models. In both models, *TP53* mutation status showed statistically significant negative impact on OS (Table 4). The number of patients who underwent HSCT was not statistically different between *TP53*-mutated and WT patients (9 patients for *TP53*-mutated [24%] versus 26 patients for *TP53* WT [20%], P = 0.62). For patients who underwent HSCT, survival outcome after HSCT was not statistically different between *TP53*-mutated and WT patients (median OS after HSCT, *TP53*-mutated vs. WT, 6.3 months vs. 8.8 months, P = 0.95).

**Table 4:** Multivariate analysis for overall survival in MDS patients treated with HMA therapy

Model 1				Reduced Model		
	HR	95% CI	P –value	HR	95% CI	P –value
**TP53 mutation (mutated vs. WT)**	3.31	1.20-9.08	0.02	3.01	1.58-5.69	0.0007
**IPSS-R risk (high/very high vs. very low/low/intermediate)**	2.24	1.04-4.83	0.04	2.31	1.08-4.94	0.03
**Therapy-related (yes vs. de novo)**	1.79	0.98-3.28	0.06			
**Monosomal karyotype (monosomal vs. non-monosomal)**	0.86	0.31-2.40	0.77			
**Model 2**						
	**HR**	**95% CI**	**P –value**			
**TP53 mutation (mutated vs. WT)**	3.66	1.53-8.75	0.004			
**IPSS-R risk (high/very high vs. very low/low/intermediate)**	2.35	1.08-5.12	0.03			
**Therapy-related (yes vs. de novo)**	1.77	0.97-3.23	0.06			
**Complex karyotype (complex vs. non-complex)**	0.73	0.3-1.75	0.47			

### Longitudinal follow up of TP53 mutation

**Figure 5:**
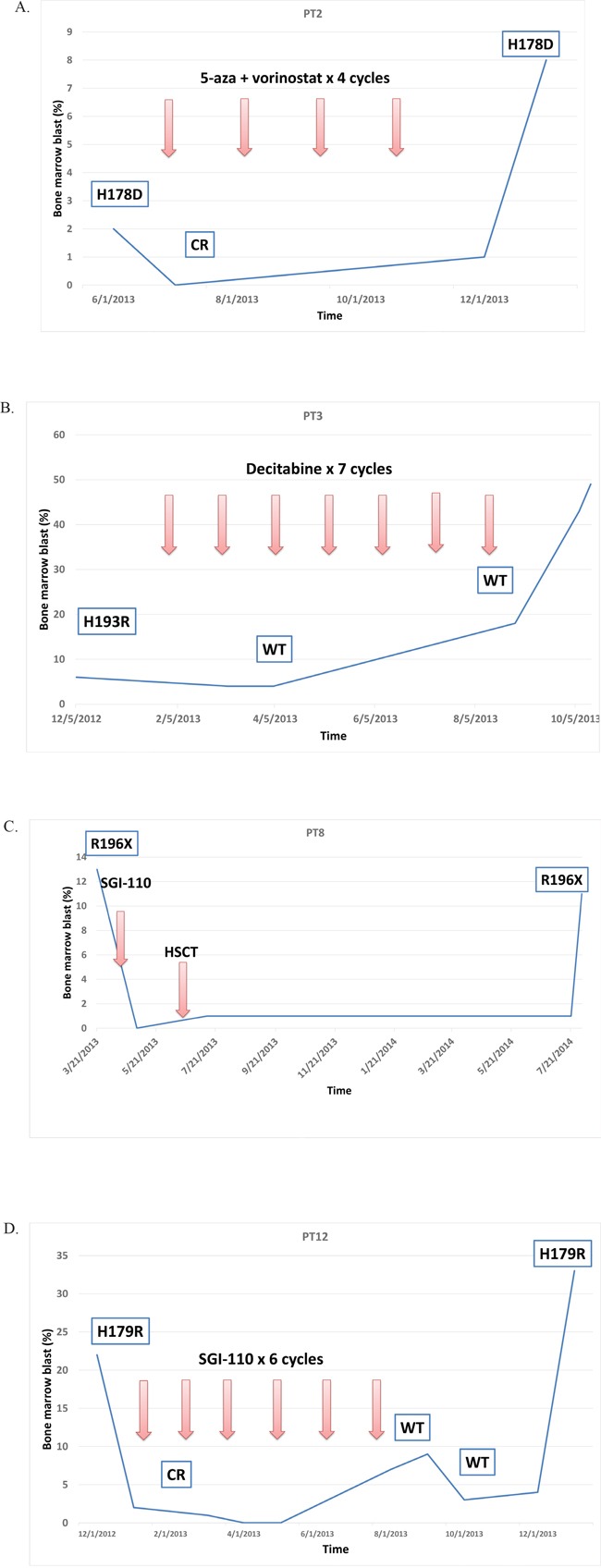
Representative cases of longitudinal *TP53* follow up Except for PT3 case, the same *TP53* mutations persisted at times of disease progression or relapse. **A.** The patient PT2 had *TP53* p.H178D mutation. Received 4 cycles of 5-azacitidine and vorinostat achieving complete response (CR). When disease progressed, the same *TP53* p.H178D mutation was detected. **B.** The patient PT3 had *TP53* p.H193R mutation Received 7 cycles of standard of care decitabine and after 3 cycles, *TP53* mutation became negative on bone marrow. When disease transformed to AML, *TP53* was still wild type (WT). **C.** The patient PT8 had *TP53* p.R196* nonsense mutation and received 1 cycle of guadecitabine (SGI-110) followed by allogeneic hematopoietic stem cell transplant (HSCT). One year later, when disease relapsed, the same *TP53* p.R196* mutation was detected in bone marrow. **D.** The patient PT12 had *TP53* p. H179R mutation. Received 6 cycles of guadecitabine (SGI-110) and achieved CR. *TP53* sequencing showed WT. However, when disease relapsed, the same *TP53* p. H179R was detected in bone marrow.

Of the 38 patients with *TP53* mutations, 13 had at least one longitudinal sample sequenced for *TP53* mutations after HMA therapy. Clinical course and *TP53* mutation follow-up of these patients is summarized in Figure 5 and Supplemental Figure 3. Seven of 13 patients achieved initial CR with HMA therapy. All 7 eventually lost response and progressed and at progression all 7 of these patients were identified to have the same *TP53* mutations. Two patients who underwent HSCT later relapsed. At the time of relapse, the same *TP53* mutations were also detected in those patients' bone marrow. Overall, except for two patients, the same *TP53* mutations were persistently detected at the time of disease progression or relapse.

## DISCUSSION

In this study, we investigated mutational landscape, clinical correlation, prognostic and predictive value of *TP53* mutations in MDS patients who were treated with HMA therapy. Several important findings emerged from this study.

First, we confirmed the strong independent prognostic significance of *TP53* mutations in MDS. Because *TP53* mutations are strongly associated with other poor prognostic factors, such as complex karyotypes, monosomal karyotype, and therapy-related disease, their independent prognostic value has been debated. In this study, *TP53* mutation status identified a distinct prognostic group among patients with complex karyotypes or IPSS-R-defined very high-risk patients. In multivariate analysis for OS, the hazard ratio of *TP53* mutation was the strongest among other predictors of poor prognosis, such as complex karyotype and IPSS-R high/very high risk. Similar findings were confirmed in AML patients with complex karyotype. [12] Taken together, these findings suggest that screening for *TP53* mutations in addition to conventional karyotyping helps identifying the highest risk groups of MDS patients. Although, majority of *TP53* mutated cases already have other poor prognostic markers and additional impact on prognosis is somewhat limited.

Second, despite their poor prognosis, *TP53*-mutated MDS patients responded to HMA therapy as well as WT patients. This is in contrast to the resistance observed in patients with *TP53*-mutated AML and MDS when they are treated with cytarabine-based cytotoxic chemotherapy. [11, 12] Recently, Bejar et al. analyzed the association between various somatic mutations and response to HMA therapy in MDS. They found that *TET2* mutations were associated with favorable response to HMA therapy but *TP53* mutations did not predict response to HMA therapy. [3] Our data confirms that *TP53* mutations do not predict for HMA response.

Despite equivalent response to HMA therapy, duration of response was significantly shorter in *TP53*-mutated patients compared to WT patients. The median response duration was approximately 6 months in *TP53*-mutated patients, and virtually all patients who responded to HMA therapy lost response within 10 months. In a subset of patients who had longitudinal follow-up for *TP53* mutations, the same *TP53* mutations almost always persisted at the time of disease progression. These findings suggest that despite its efficacy, HMA therapy is not capable of eliminating abnormal hematopoietic clones with *TP53* mutations. Similar findings of persistent *TP53* mutations after therapy have also been reported in cases with 5q- syndrome. [5]

From a clinical perspective, our findings do not discourage the use of HMA therapy in *TP53*-mutated cases but rather support its use. Considering that *TP53* mutations have been associated with resistance to cytarabine based chemotherapy [11, 12], better response is expected with HMA therapy. However, based on our findings, most responders will lose their responses within the first year of therapy. A novel strategy to extend response is clearly needed for *TP53*-mutated MDS patients. Unfortunately, current HSCT strategies have not proven to be the answer as recent studies have indicated that *TP53*-mutated MDS patients have dismal outcomes after HSCT [22], although in our limited number of patients who underwent HSCT, survival after HSCT was not different between *TP53*-mutated and WT patients. Patients did not receive pre-HSCT HMA therapy in the previous study and all of our studied patients received HMA therapy prior to HSCT, so more analysis may be needed to fully understand the outcomes of *TP53*-mutated MDS patients who receive HSCT. Nevertheless, understanding the molecular mechanism of acquired resistance to HMA therapy and developing novel therapeutic strategies are urgently needed to improve outcomes for *TP53*-mutated MDS patients.

We note that some of the findings of our study are in opposition to previous studies. Notably, we did not see favorable response rate to HMA therapy in *TET2* mutated patients. Only a part of our patients were sequenced for *TET2*, which may have decreased statistical power. In addition, although we strictly followed the response criteria defined by IWG, treatment response evaluation in MDS sustains some subjectivity. Further, there is a significant heterogeneity in the treatment regimen in our study cohort, both of which may have biased the result. Of note, even in the prior study, association between *TET2* mutations and HMA response was not robust and statistical significance became apparent only when *TET2* mutations were restricted to VAF > 10%. [3] These results suggest that application of molecular data to treatment decision-making still requires caution in MDS patients.

In summary, our study suggests that *TP53* mutation status is the strongest predictor of prognosis in MDS patients treated with HMA therapy. Its prognostic value is significant after adjusting prognostic effect of other factors such as complex karyotypes and IPSS-R risk. Despite the poor prognosis, *TP53*-mutated MDS patients respond equally as well to HMA therapy as WT *TP53* patients. However, their duration of response is significantly shorter, and *TP53* mutations almost always persist at the time of disease progression. Novel therapeutic strategies to improve duration of response in *TP53*-mutated MDS are urgently needed.

## MATERIALS AND METHODS

### Studied patients and treatment

We identified 321 patients with previously untreated MDS who were referred to The University of Texas MD Anderson Cancer Center (MDACC) between 2012 and 2014. Of the 321 patients, 168 patients (52%) were treated with HMA therapy and were therefore eligible for further analysis. Diagnosis of MDS was classified using the WHO classification system. [15] We also included patients in this study that were historically classified as refractory anemia with excess blasts in transformation (RAEB-T). [16] This is because HMA therapy has been recognized as one of the standard care for this subgroup of patients. [13] Cytogenetic and overall prognostic risks were calculated by Revised International Scoring System (IPSS-R). [17] Although IPSS-R was generated based on the data from *de novo* MDS patients, we also applied IPSS-R to therapy-related MDS patients in the current study because previous studies have shown that IPSS-R retains power in this group of patients. [18]

Seventy-eight patients (46%) received standard of care (SOC) 5-azacitidine or decitabine (38 patients received 5-azacitidine alone, and 40 patients received decitabine alone), 79 patients (47%) received either 5-azacitidine or decitabine in combination with other investigational agents under various clinical trials (68 patients received 5-azacitidine combinations, and 11 patients received decitabine combinations), and 11 patients (7%) received guadecitabine (SGI-110) [19] as a single agent under a clinical trial. Details of therapy regimens are described in Supplemental Table 1. The median interval from original diagnosis at outside institutions to presentation to our institution was 0.6 months (range: 0-62 months). All bone marrow samples analyzed in this study were obtained at the time of presentation to MDACC. Written informed consent was provided by all studied patients, and the study protocol was approved by the institutional review board (IRB) at MD Anderson Cancer Center. The study was conducted in accordance with the Declaration of Helsinki.

### Sample processing, DNA sequencing, and variant calling

*TP53* analysis in all 168 patients was performed on the initial bone marrow sample by one of the following methods: whole-exome sequencing (WES, N = 53), targeted gene capture deep sequencing using a next-generation sequencing (NGS) platform (28-gene panel, N = 26 or 53-gene panel, N = 89). For WES, Agilent SureSelect All Exon V4 was used for exome capture hybridization, and an Illumina HiSeq 2000 sequencer was used for sequencing with 75 base pair paired-end reads. Both the 28-gene and 53-gene panel sequencing was performed on Illumina MiSeq platform as previously described. [20] The panels of genes sequenced by the 28- and 53-gene panels are listed in Supplemental Table 2. WES and the 28-gene panel covered the entire coding sequence of *TP53*, and the 53-gene panel covered the entire coding sequence of exons 4-8 and part of exons 2 and 10. The median coverage within the targeted region was 124x and 4,000x with WES and targeted gene capture deep sequencing, respectively. Some of the *TP53*-mutated patients had longitudinal assessment of *TP53* mutations by polymerase chain reaction (PCR)-based Sanger sequencing. Methods for variant calling and filtering process are described in Supplemental Method.

### Definition of response and survival outcome

Definition of response to HMA therapy followed the 2006 International Working Group (IWG) criteria. [21] Overall response (OR) was defined as having either complete response (CR), partial response (PR), or hematological improvement (HI). Duration of response was calculated from the time of response to the time of loss of response or last follow-up, whichever occurred first. Patients who underwent hematopoietic stem cell transplant (HSCT) were censored at the time of transplant when calculating response duration. Time to achieve best response (TTR) was calculated from the date of HMA therapy initiation to the date of best response. Overall survival (OS) was calculated from the date of HMA therapy initiation to death or the last follow-up date.

### Statistical methods

The chi-square or Fisher exact test was used to assess differences in categorical variables, and the Mann-Whitney *U* test was used to analyze continuous variables difference. The log-rank test was used to examine between-group differences in survival outcome. Multivariate analysis was conducted using Cox proportional hazards regression. Statistical analyses were performed using SPSS (version 22; IBM Corporation, Armonk NY).

## SUPPLEMENTARY MATERIALS AND METHODS, REFERENCES, TABLES AND FIGURES


