# Supplementary material for: Clinical implications of TP53 mutations in myelodysplastic syndromes treated with hypomethylating agents
Source: Oncotarget. 2016 Feb 9;7(12):14172–87. doi: 10.18632/oncotarget.7290 (PMC4924706; doi:10.18632/oncotarget.7290)
Supplement: Supplementary file 1 [file oncotarget-07-14172-s001.pdf]

SUPPLEMENTARY MATERIALS AND METHODS

Variant calling and filtering

For variant calling, sequencing data were aligned to the hg19 human genome reference using Burrows-Wheeler Aligner (BWA) followed by mark duplication, in-del realignment, and base recalibration using GATK best practices tools (https://www.broadinstitute.org/gatk/guide/best-practices?bpm=DNAseq). [1] The resulting BAM files were preprocessed, and base substitutions and small insertions/deletions were called using Mutect and Pindel, respectively, against an unmatched normal sample, as previously described. [2-4] The called variants were annotated using ANNOVAR and then filtered for potential single nucleotide polymorphisms (SNPs) based on the dbSNP (http://www.ncbi.nlm.nih.gov/SNP/), 1000 genome project (http://www.1000genomes.org/), and ESP 6500 (http://evs.gs.washington.edu/EVS/) databases. [5]

SUPPLEMENTARY REFERENCES

1. Li H, Durbin R: Fast and accurate short read alignment with Burrows-Wheeler transform. Bioinformatics 25:1754-60, 2009
2. Cibulskis K, Lawrence MS, Carter SL, et al: Sensitive detection of somatic point mutations in impure and heterogeneous cancer samples. Nat Biotechnol 31:213-9, 2013
3. Ye K, Schulz MH, Long Q, et al: Pindel: a pattern growth approach to detect break points of large deletions and medium sized insertions from paired-end short reads. Bioinformatics 25:2865-71, 2009
4. Takahashi K, Roh W, Zhang J, et al: Clonal evolution of acute myeloid leukemia relapsed after 19 years of remission. Am J Hematol 90:E134-5, 2015
5. Wang K, Li M, Hakonarson H: ANNOVAR: functional annotation of genetic variants from high-throughput sequencing data. Nucleic Acids Res 38:e164, 2010

SUPPLEMENTARY FIGURES AND TABLES

Supplementary Table S1: Summary of the HMA therapy regimen that 168 patients with MDS/CMML received upfront

Therapy regimen	N = 168 (%)
Azacitidine SOC	38 (23)
Decitabine SOC	40 (24)
Azacitidine + Birinapant	1 (<1)
Azacitidine + GM-CSF	3 (2)
Azacitidine + Lenalidomide	20 (12)
Azacitidine + Panobinostat	1 (<1)
Azacitidine + PKC412	1 (<1)
Azacitidine + Pracinostat	18 (11)
Azacitidine + Rigorsetib	1 (<1)
Azacitidine + Ruxolitinib	4 (2)
Azacitidine + Vorinostat	19 (11)
Decitabine + Sapacitabine	1 (<1)
Decitabine + Clofarabine	3 (2)
Decitabine + Vosaroxin	7 (4)
Guadecitabine (SGI-110)	11 (7)

Supplementary Table S2: List of genes sequenced by either 28 gene panel or 53 gene panel NGS platform. Genes overlapped between the 2 methods are underlined

28 gene panel		53 gene panel			
ABL1	*KRAS*	*ABL1*	*FBXW7*	*KIT*	*SMAD4*
ASXL1	*MDM2*	*AKT1*	*FGFR1*	*KLHL6*	*SMARCB1*
BRAF	*MLL*	*ALK*	*FGFR2*	*KRAS*	*SMO*
DNMT3A	*MPL*	*APC*	*FGFR3*	*MET*	*SRC*
EGFR	*MYD88*	*ATM*	*FLT3*	*MLH1*	*STK11*
EZH2	*NOTCH1*	*BRAF*	*GNA11*	*MPL*	*TP53*
FLT3	*NPM1*	*CDH1*	*GNAQ*	*NOTCH1*	*VHL*
GATA1	*NRAS*	*CDKN2A*	*GNAS*	*NPM1*	*XPO1*
GATA2	*PTPN11*	*CSF1R*	*HNF1A*	*NRAS*	
HRAS	*RUNX1*	*CTNNB1*	*HRAS*	*PDGFRA*	
IDH1	*TET2*	*DNMT3A*	*IDH1*	*PIK3CA*	
IDH2	*TP53*	*EGFR*	*IDH2*	*PTEN*	
IKZF2	*WT1*	*ERBB2*	*JAK2*	*PTPN11*	
JAK2		*ERBB4*	*JAK3*	*RB1*	
KIT		*EZH2*	*KDR*	*RET*	

Supplementary Table S3: Treatment response by *TP53* mutation status in a subgroup of patients treated with standard of care HMA or HMA combination with investigational agents

		CR	no CR	P	OR	no OR	P
SOC HMA	*TP53* mutated	4	7	0.39	5	6	0.25
	TP53 WT	18	48		20	46	
HMA combination	*TP53* mutated	9	18	0.62	11	16	0.47
	TP53 WT	18	46		21	43	

Supplementary Table S4: Association between treatment response and various myeloid driver mutations

Mutation (/tested)	Mutation rate (%)	CR rate (%, mutated vs. WT)	P	OR rate (%, mutated vs. WT)	P
ASXL1 (/79)	17	23% vs. 33%	.35	23% vs. 36%	.28
BCOR/BCORL1 (/53)	6	67% vs. 32%	.26	67% vs. 36%	.32
CBL (/53)	13	43% vs. 32%	.45	43% vs. 37%	.54
CUX1 (/53)	6	33% vs. 67%	.74	33% vs. 38%	.68
DNMT3A (/168)	6	10% vs. 30%	.16	10% vs. 35%	.09
EZH2 (/168)	2	33% vs. 29%	.65	3% vs. 34%	.73
IDH1 (/168)	3	40% vs. 29%	.46	40% vs. 34%	.55
IDH2 (/168)	6	30% vs. 29%	.60	30% vs. 34%	.54
KRAS (/168)	4	17% vs. 30%	.44	17% vs. 34%	.34
NRAS (/168)	4	0% vs 30%	.09	0% vs. 35%	.05
PTPN11 (/168)	4	43% vs. 29%	.33	43% vs. 34%	.44
RUNX1 (/79)	20	25% vs. 33%	.38	25% vs. 37%	.29
SF3B1 (/53)	4	50% vs. 33%	.57	50% vs. 37%	.62
SRSF2 (/53)	11	17% vs. 36%	.33	17% vs. 40%	.26
TET2 (/79)	23	17% vs. 36%	.10	22% vs. 38%	.18
U2AF1 (/53)	13	29% vs. 35%	.56	29% vs. 39%	.47
ZRSR2 (/53)	6	0% vs. 36%	.28	0% vs. 40%	.23
KRAS/NRAS (/168)	8	8% vs. 31%	.06	8% vs. 36%	.03
IDH1/IDH2 (/168)	21	33% vs. 29%	.46	33% vs. 34%	.60
Splicing gene (/53)	34	22% vs 40%	.16	22% vs. 46%	.08
TET2+/ASXL1+ (/79)	4	0% vs. 33%	.31	0% vs. 36%	.28
TET2+/ASXL1- (/79)	19	20% vs. 34%	.23	27% vs. 36%	.36

Supplementary Table S5: Univariate analysis for overall survival

Variables	Median OS (95%CI)	P value
**Age > 70 y (vs. ≤ 70 y)**	13.3 (11.7-14.8) vs. 16.0 (9.0-22.9)	0.30
**RAEB-T (vs. others)**	13.1 (11.8-17.7) vs. 16.0 (11.6-14.5)	0.55
**Therapy-related (vs. de novo)**	9.0 (5.9-vs. 12.1) vs. 16 (11.3-20.7)	0.02
**Complex karyotype (vs. others)**	10.9 (8.8-12.9) vs. 20.1 (14.0-26.2)	<0.001
**ANC < 0.8 x 10^9/L (vs. ≥ 0.8 x 10^9/L)**	12.3 (11.7-14.9) vs. 18 (11.0-24.9)	0.77
**HGB < 8 g/dL (vs. ≥ 8 g/dL)**	10.6 (3.6-17.5) vs. 14.8 (10.6-18.9)	0.17
**PLT < 50 x 10^9/L (vs. ≥ 50 x 10^9/L)**	12.9 (10.4-15.6) vs. 18.0 (12.2-23.7)	0.12
**BM blast >10% (vs. ≤ 10%)**	13.3 (12.1-14.5) vs. 14.8 (11.6-17.9)	0.65
**Monosomal karyotype (vs. others)**	10.5 (7.4-13.6) vs. 20.7 (16.4-24.9)	<0.001
**IPSS-R high/very high risk (vs. others)**	12.9 (10.7-15.1) vs. 20.7 (1.4-40.3)	0.001
**HSCT (vs. no HSCT)**	14.2 (9.8-18.7) vs. 14.7 (9.6-19.8)	0.30
***ASXL1* mutated (vs. WT)**	NR vs. 14.3 (11.2-17.4)	0.78
***CBL* mutated (vs. WT)**	6.13 (NR) vs. 13.3 (11.9-14.7)	0.86
***DNMT3A* mutated (vs. WT)**	11.0 (7.3-14.8) vs. 14.8 (9.4-20.2)	0.81
***EZH2* mutated (vs. WT)**	NR vs. 14.3 (11.5-17.0)	0.67
***IDH1* mutated (vs. WT)**	14.0 (0.0-29.6) vs. 14.8 (10.3-19.2)	0.76
***IDH2* mutated (vs. WT)**	22.9 (NR) vs. 14.3 (11.3-17.2)	0.74
***KRAS* mutated (vs. WT)**	13.2 (0.0-28.5) vs. 14.8 (11.5-18.0)	0.77
***NRAS* mutated (vs. WT)**	8.8 (3.1-14.6) vs. 14.8 (10.4-19.1)	0.10
***PTPN11* mutated (vs. WT)**	NR vs. 14.8 (11.8-17.3)	0.28
***RUNX1* mutated (vs. WT)**	9.7 (6.6-12.9) vs. 14.3 (10.8-17.8)	0.36
***SRSF2* mutated (vs. WT)**	6.1 (0.0-15.6) vs. 13.3 (11.4-15.2)	0.48
***TET2* mutated (vs. WT)**	13.2 (6.8-19.6) vs. 14.3 (11.2-17.4)	0.62
***U2AF1* mutated (vs. WT)**	9.7 (6.6-12.8) vs. 13.3 (11.6-15.0)	0.27
**Splicing gene mutated (vs. WT)**	21.3 (NR) vs. 13.3 (11.3-15.3)	0.65
***KRAS/NRAS* mutated (vs. WT)**	13.2 (8.8-17.6) vs. 14.8 (9.2-20.3)	0.23
***IDH1/2* mutated (vs. WT)**	14.8 (13.3-16.3) vs. 14.3 (9.9-18.6)	0.90

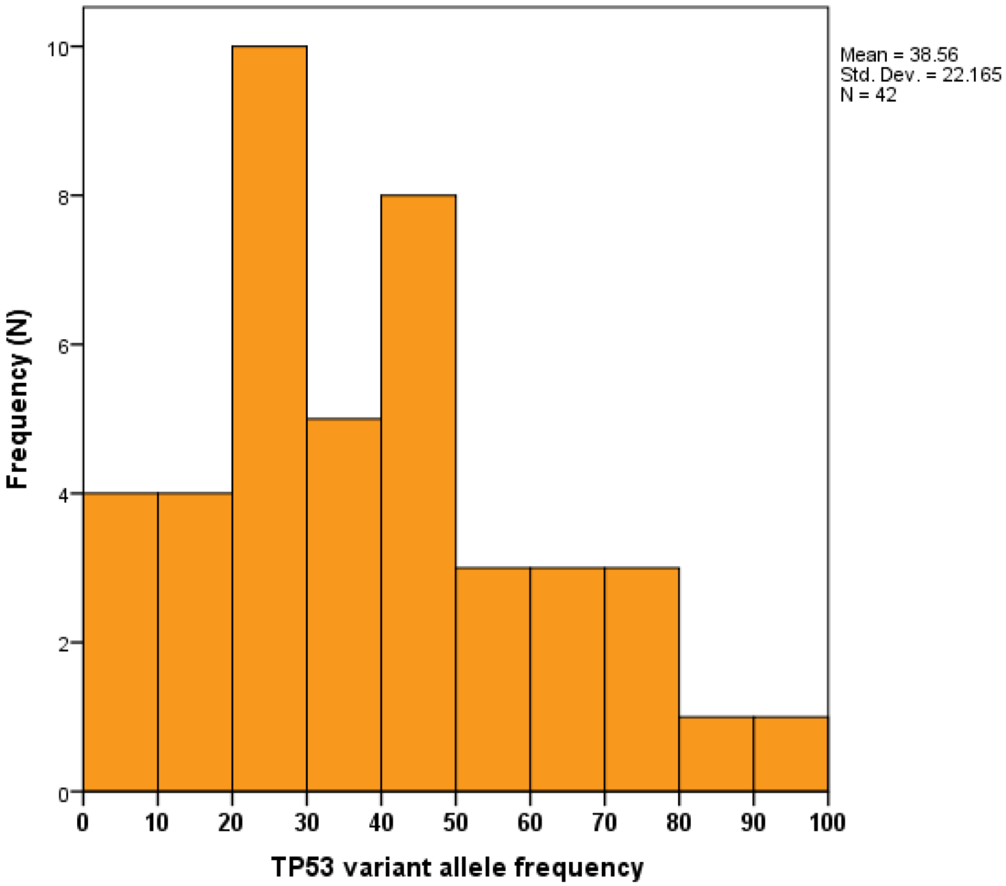

Supplementary Figure S1: Distribution of VAF for *TP53* mutations.

A.

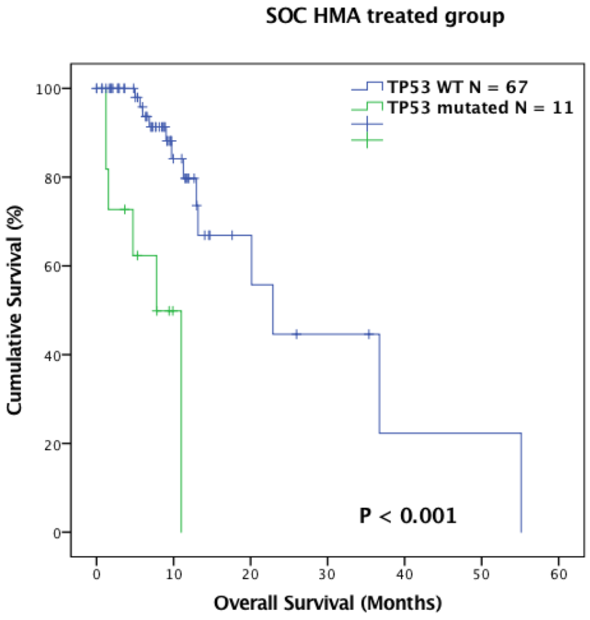

B.

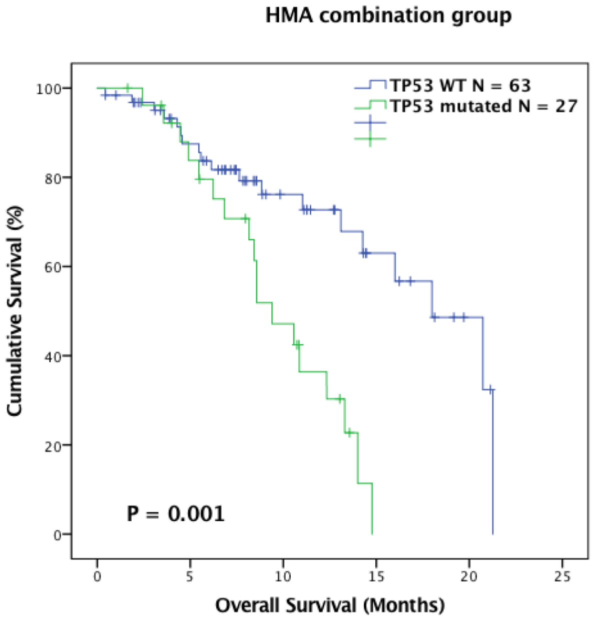

Supplementary Figure S2: Comparison of overall survival by *TP53* mutation status in A. Patients treated with SOC HMA and in B. patients treated with HMA combination.

A.

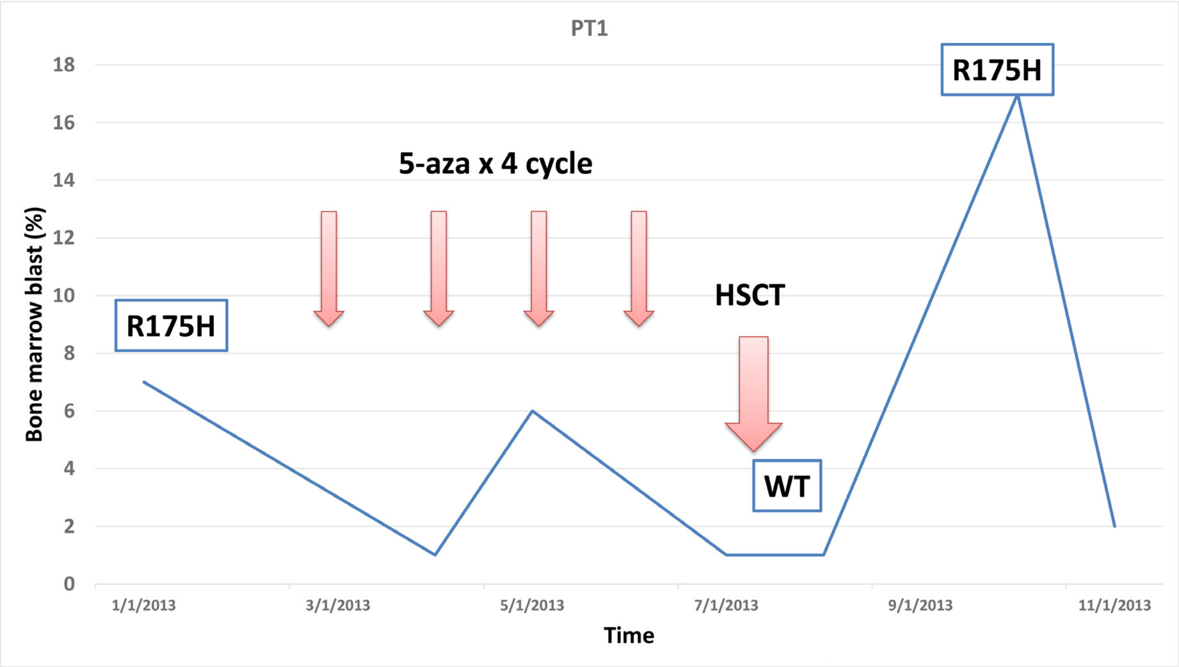

B.

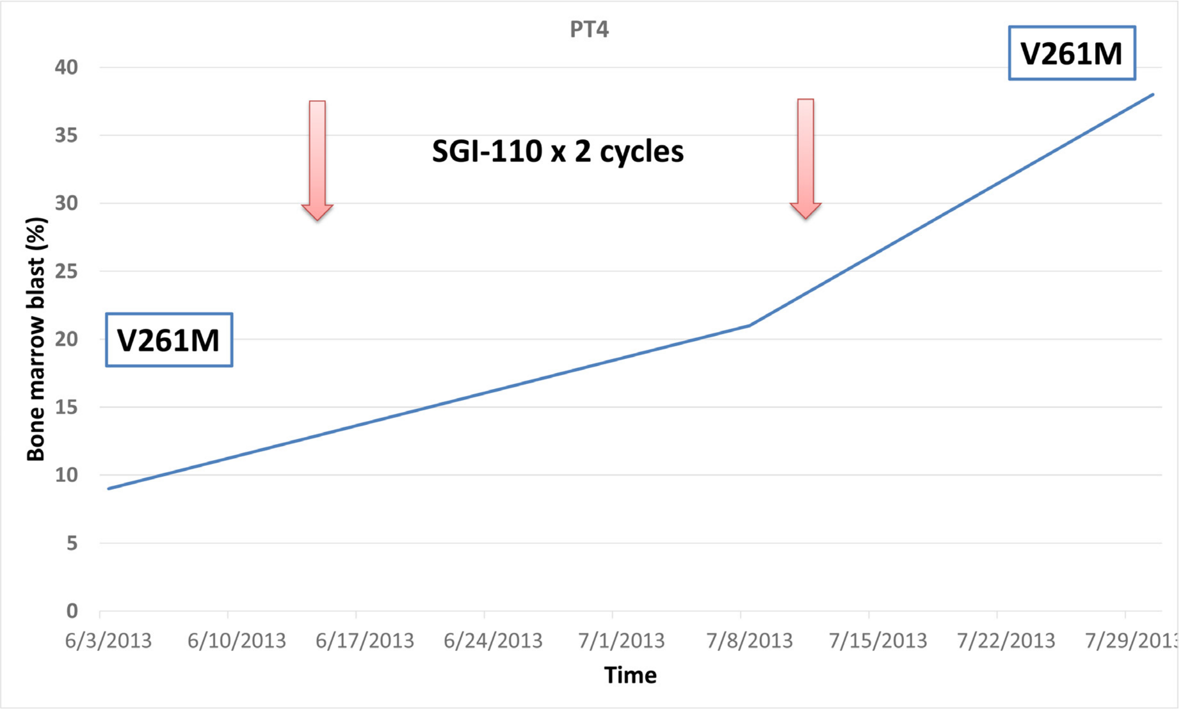

(*Continued*)

C.

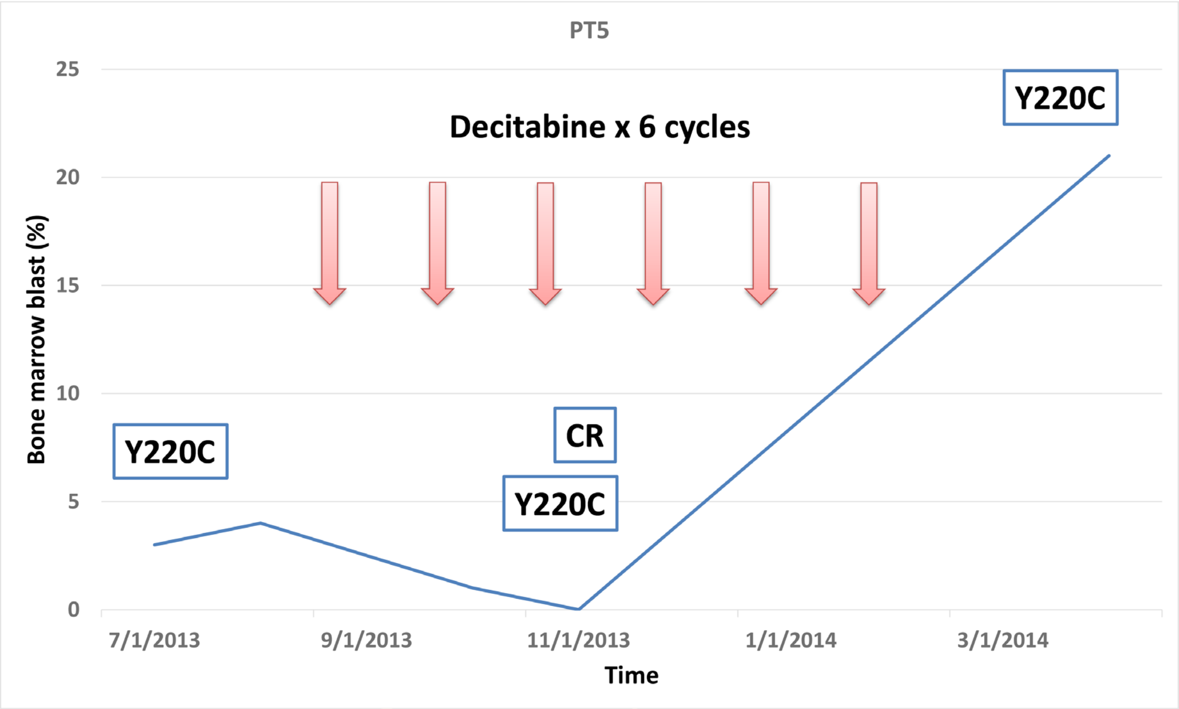

D.

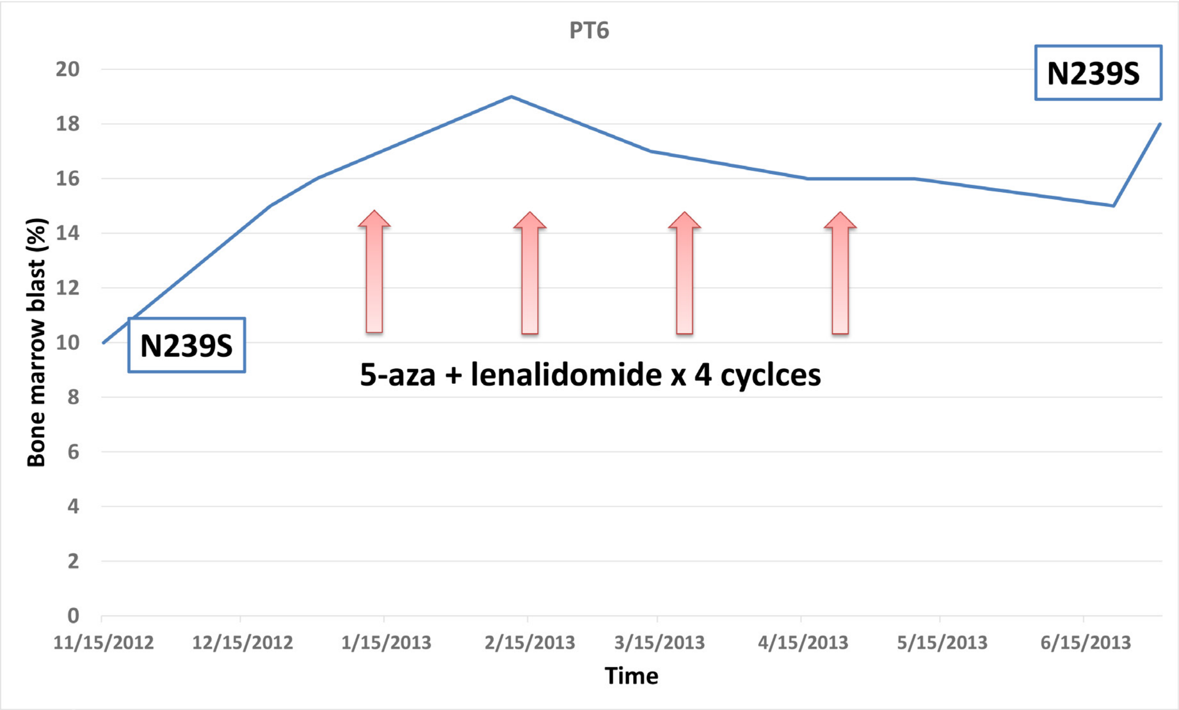

(*Continued*)

E.

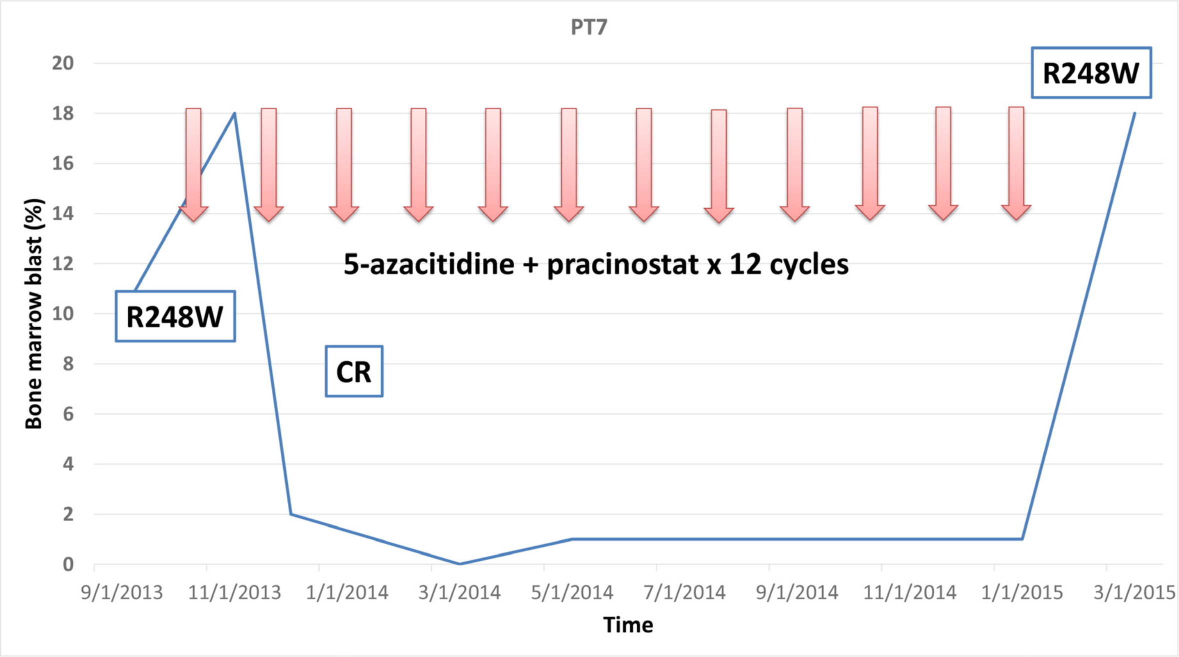

F.

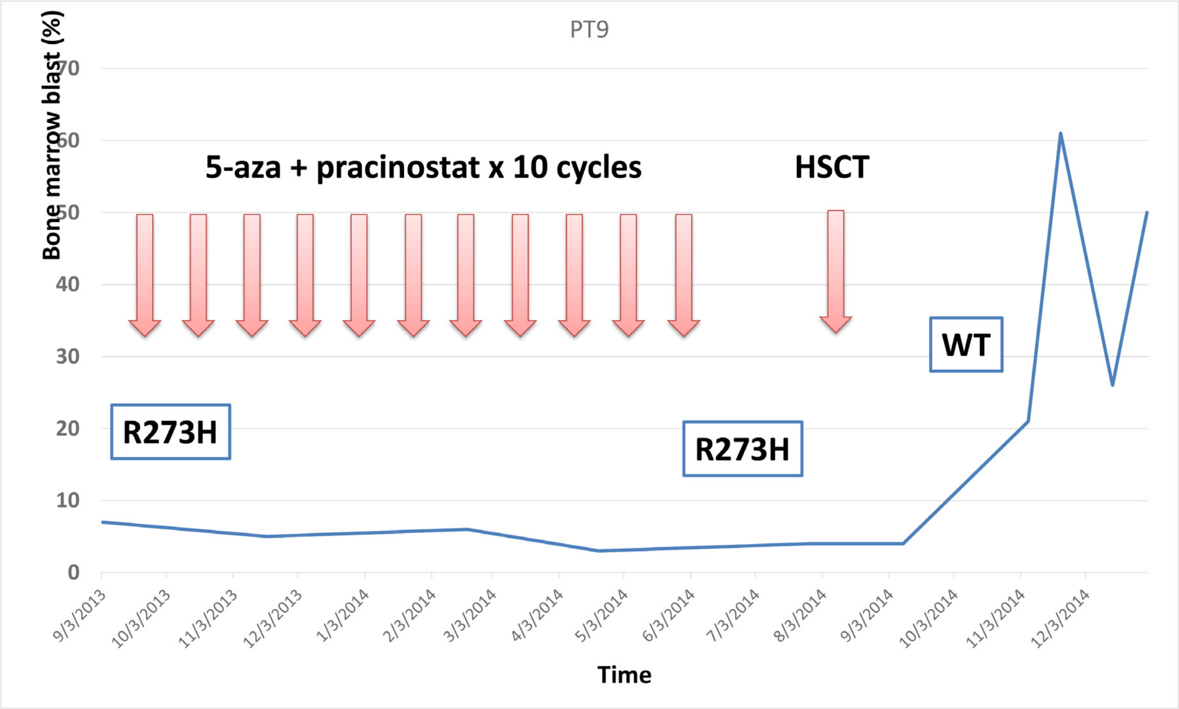

(*Continued*)

G.

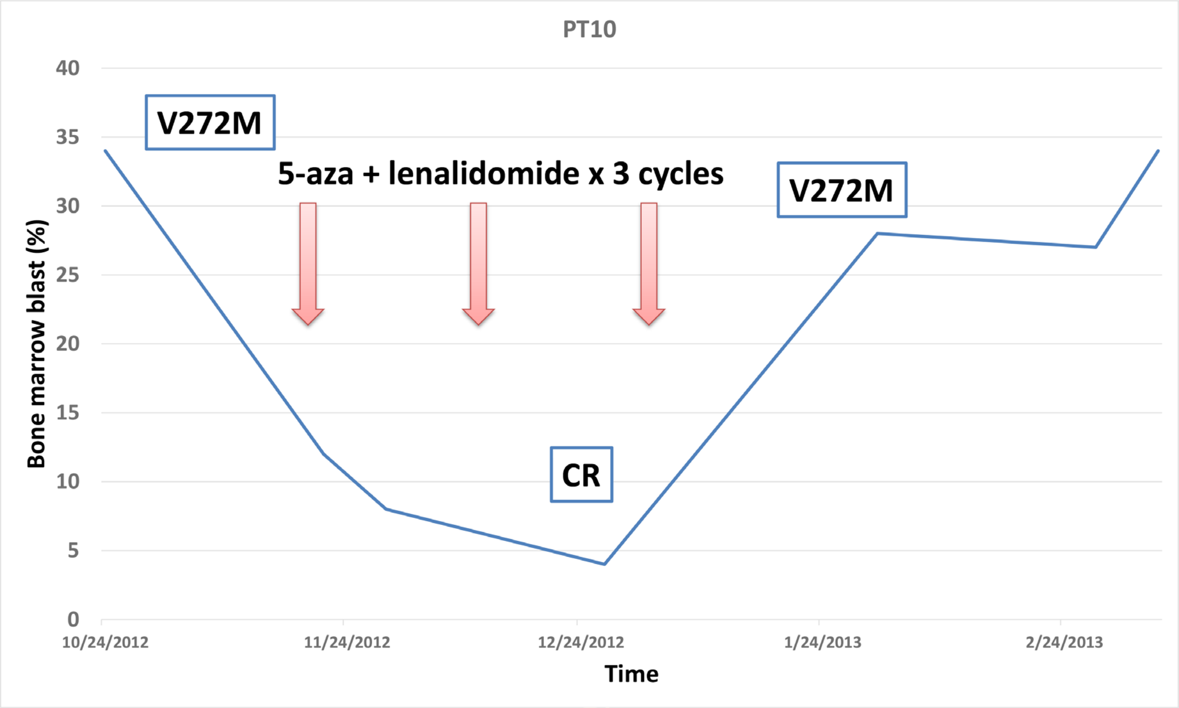

H.

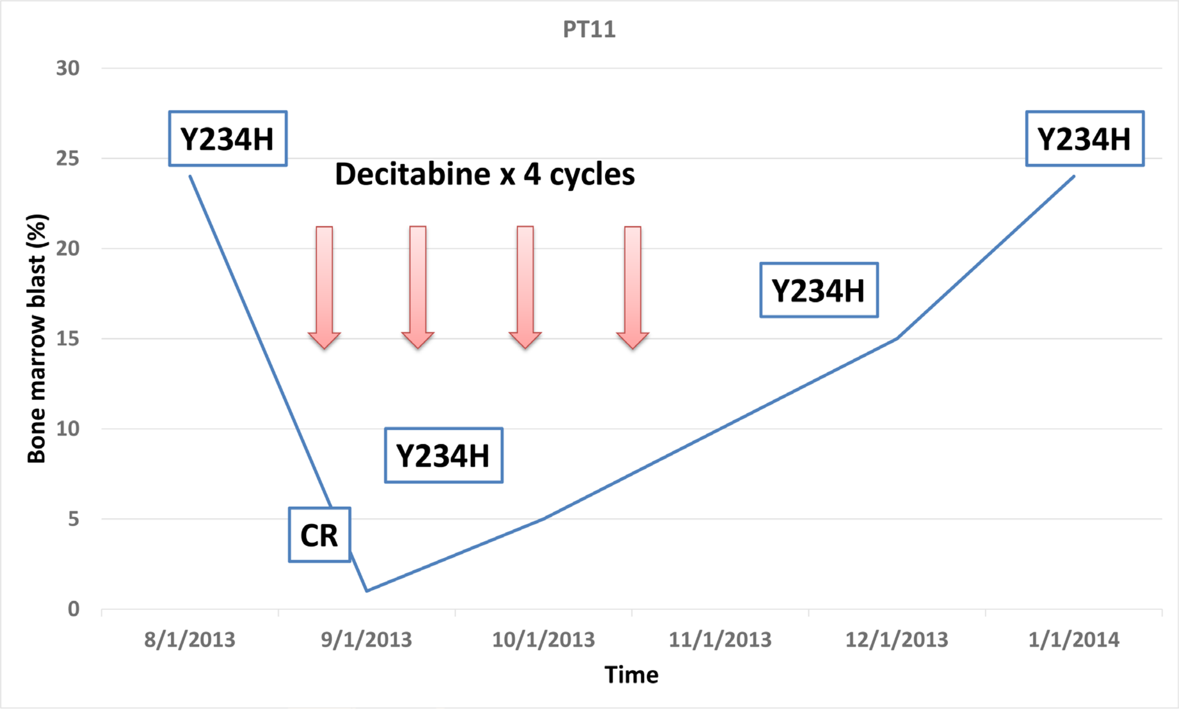

(*Continued*)

I.

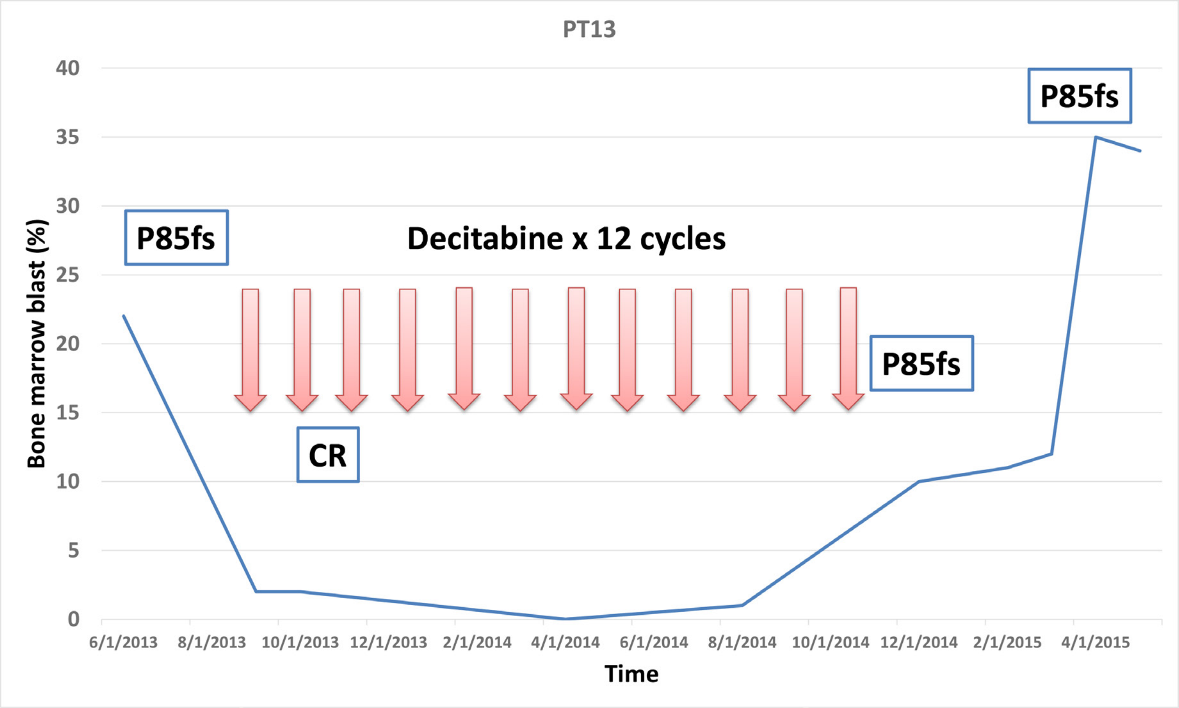

Supplementary Figure S3: Other cases with longitudinal *TP53* mutation follow up that are not listed in Figure 5.
